# Deformation Behaviors in Single BCC‐Phase Refractory Multi‐Principal Element Alloys under Dynamic Conditions

**DOI:** 10.1002/advs.202508180

**Published:** 2025-08-04

**Authors:** Chanho Lee, Deva Prasaad Neelakandan, Dongyue Xie, Juntan Li, Chia‐Yi Wu, Aomin Huang, Leeseung Kang, Shuozhi Xu, Barton C. Prorok, Dong‐Joo Kim, Marc A. Meyers, Haixuan Xu, Peter K. Liaw, Yi‐Chia Chou, Ke An, George T. Gray, Nan Li, Gian Song, Saryu J. Fensin

**Affiliations:** ^1^ Materials Engineering Auburn University Auburn AL 36849 USA; ^2^ Center for Integrated Nanotechnologies MPA Division Los Alamos National Laboratory Los Alamos NM 87545 USA; ^3^ Department of Materials Science and Engineering The University of Tennessee Knoxville TN 37996‐2100 USA; ^4^ Department of Electrophysics National Yang Ming Chiao Tung University Hsinchu 30010 Taiwan; ^5^ Department of Mechanical and Aerospace Engineering University of California San Diego CA 92093 USA; ^6^ Korea Institute for Rare Metals Korea Institute of Industrial Technology (KITECH) Incheon 21655 Republic of Korea; ^7^ School of Aerospace and Mechanical Engineering University of Oklahoma Norman OK 73019‐1052 USA; ^8^ Neutron Scattering Division Oak Ridge National Laboratory Oak Ridge TN 37831 USA; ^9^ Division of Advanced Materials Engineering Kongju National University Cheonan Chungnam 330‐717 Republic of Korea

**Keywords:** deformation twinning, dynamic deformation, refractory multi‐principal element alloys

## Abstract

The mechanical behavior and microstructural evolution of a BCC‐phase NbTaTiV refractory multi‐principal element alloy (RMPEA) is studied over a wide range of strain rates (10^−3^ to 10^3^ s^−1^) and temperatures (room temperature to 850 °C). The mechanical property of present RMPEA shows less strain‐rate dependence and strong resistance to softening at high temperatures. Under high strain‐rate loading, the formation of thin type‐I twins is observed, which could lead to an increase in strain‐hardening rates. However, this hardening mechanism competes with adiabatic heating effects, resulting in the deterrence of strain‐hardening behaviors. In contrast, substantial strain‐hardening occurs at cryogenic temperatures due to the formation of twins, which act as stronger barriers to dislocation motion and interact with each other. To further understand the different strain‐hardening behaviors, density functional theory (DFT) calculations predict relatively low stacking fault energies and high twinning stress for the NbTaTiV RMPEA.

## Graphical abstract

Exceptional mechanical stability of refractory multi‐principal element alloy (RMPEA) across various strain‐rates and temperature is studied through multiscale experiments coupled with theoretical calculations. This stability originates from competition between twinning and adiabatic heating during dynamic deformation, contributed from severe lattice distortion and edge dislocation strengthening. However, cryogenic testing still shows pronounced strain hardening from abundant twins.

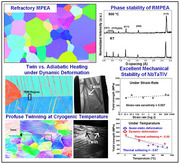

## 1 Introduction

Multi‐principal element alloys (MPEA) have drawn substantial attention due to their outstanding mechanical properties beyond traditional metallic materials.^[^ ^1^, ^2^, ^3^, ^4^, ^5^, ^6^, ^7^, ^8^, ^9^ ^]^ MPEAs typically contain multiple elements in near‐equiatomic ratios, which in turn, lead to a high configurational entropy and locally strained atomic structure in a single solid‐solution phase. These features enhance properties such as excellent single‐phase stability, high strength, and resistance to softening at high temperatures.^[^ ^10^, ^11^, ^12^, ^13^, ^14^ ^]^ Owing to these unique properties, numerous efforts have been made to design and develop MPEA systems for extreme environmental applications (high temperatures and pressures). This effort has specially focused on the development of refractory MPEAs (RMPEAs), which are composed of refractory elements (such as Nb, Mo, Ta, W, Ti, V, Cr, Zr, and Hf) and form a single body‐centered‐cubic (BCC)‐phase solid‐solution with high yield strength at temperatures above 800 °C.^[^ ^13^, ^15^, ^16^, ^17^, ^18^, ^19^, ^20^, ^21^ ^]^ Hence, RMPEAs are rapidly emerging as top candidates for high‐temperature applications.

To make these alloys more desirable for applications, including aeronautical engineering, automotive industry, and marine engineering, it is imperative to study their mechanical behavior as a function of strain‐rate. Hence, there has been an increasing interest in exploring the microstructural evolution and mechanical behavior of these MPEAs at high strain‐rates and temperatures. For example, Kumar et al. investigated the high‐strain deformation behavior of face‐centered‐cubic (FCC) Al_0.1_CoCrFeNi MPEA and found that the yield strength increases by approximately 60% with a significant increase in the rate of strain‐hardening at a strain‐rate of 2 × 10^3^ s^−1^, compared to its behavior at quasi‐static rates.^[^ ^22^ ^]^ The substantial strain‐hardening and ductility were attributed to profuse twinning due to the low stacking‐fault energy of this MPEA. Li et al. further studied the mechanical response and microstructural evolutions of Al_0.3_CoCrFeNi MPEA at strain rates varying from 10^−4^ to 10^3^ s^−1^.^[^ ^23^ ^]^ This FCC‐phase MPEA also showed a remarkable increase in the yield strength at a strain rate of 10^3^ s^−1^, indicating a significant strain‐rate sensitivity (0.053). The strain‐hardening rate of the Al_0.3_CoCrFeNi MPEA was also found to be higher than pure aluminum,^[^ ^24^ ^]^ which is usually enabled by solid‐solution hardening, dislocations, and twinning. He et al.,^[^ ^25^ ^]^ Wang et al.,^[^ ^26^ ^]^ and Li et al.,^[^ ^27^ ^]^ reported the mechanical properties of the CoCrFeMnNi (Cantor MPEA) at a wide range of strain rates from 10^−4^ to 2 × 10^3^ s^−1^. Similar to Al_x_CoCrFeNi MPEAs,^[^ ^22^, ^23^ ^]^ the Cantor MPEA also showed strong strain‐rate dependency of yield strength with a high value of strain‐rate sensitivity (0.022). Importantly, twinning was found to be the dominant deformation mechanism at these high strain rates leading to an increase in strain‐hardening rate.

In contrast to the abundance of studies on FCC‐phase MPEAs at high strain rates, limited studies have explored the mechanical behaviors of BCC‐phase RMPEAs under dynamic loading. For instance, Dirras et al.^[^ ^28^ ^]^ and Hu et al.^[^ ^29^ ^]^ have studied microstructural evolution and mechanical behavior in the BCC‐phase HfNbTaTiZr RMPEA at various strain rates of 10^−3^ to 3 × 10^3^ s^−1^ at elevated temperatures. It was found that its yield strength also increases with strain rate, but the magnitude of increase was smaller in comparison to the FCC‐phase MPEAs, indicating a lower strain‐rate dependence on yield strength. Furthermore, the strain‐hardening rate of RMPEAs at a strain rate of 10^3^ s^−1^ was found to be lower than the quasi‐static one at all temperature ranges tested [from room temperature (RT) to 600 °C]. Additionally, Zhang et al. further investigated the relationship between strain rate and the deformation behavior in the HfNbTaTiZr RMPEA with the addition of Mo (HfNbTaTiZrMo*_x_*).^[^ ^30^ ^]^ The yield strength trend also exhibited a dependence on the strain rate. However, less strain‐hardening at strain rates of 2 × 10^3^‐6 × 10^3^ s^−1^ was found for both the HfNbTaTiZrMo_x_ and HfNbTaTiZr RMPEAs in comparison to quasi‐static rates.^[^ ^28^, ^29^, ^30^ ^]^ Moreover, atomistic simulations by Xu et al. found that the strength of HfMoNbTaTi is less dependent on the strain rate compared with Mo and Nb.^[^ ^31^ ^]^

Recently, Lee et al. designed and developed a single BCC‐phase NbTaTiV RMPEA, which showed desirable mechanical properties at RT.^[^ ^3^, ^4^, ^32^, ^33^ ^]^ The quasi‐static mechanical testing showed a high yield strength of 1,273 MPa without fracturing the sample until a 30% strain. The high strength of NbTaTiV was mainly attributed to solid‐solution strengthening, which stemmed from the severely distorted crystalline lattice.^[^ ^34^, ^35^ ^]^ The mechanical stability of the NbTaTiV RMPEA at elevated temperatures has been further studied considering single‐crystal elastic moduli.^[^ ^33^ ^]^ The mechanical stability was assessed by Cauchy pressure, Pugh ratio, and Poisson's ratio, indicating that the NbTaTiV is intrinsically ductile at elevated temperatures. In the present study, we have systematically conducted uniaxial compression tests on the NbTaTiV RMPEA at elevated temperatures and various strain rates ranging from 10^−3^ to 10^3^ s^−1^ to investigate the effect of strain rate and temperature on its mechanical behavior and mechanism evolutions. This study integrates the analysis of deformation mechanisms using both experimental efforts coupled with DFT modeling. Neutron diffraction (ND) measurements were performed to assess the phase stability at elevated temperatures. Secondary electron microscope (SEM) equipped with electron back‐scattered diffraction (EBSD) was used for further characterization of microstructure and defects. EBSD–inverse pole figure (IPF) maps were used to characterize the grains as well as the deformation twins. Regions of deformation twins were selected for further analysis using transmission electron microscopy (TEM). Diffraction patterns were obtained from the deformation bands to determine the type of twins formed. Scanning transmission electron microscopy (STEM) was used to determine the types of dislocations generated in the present RMPEA. DFT calculations were performed to obtain values of stacking‐fault energies and energy required for nucleation of twins in the present RMPEA.

## 2 Results

### 2.1 Microstructure of NbTaTiV RMPEA

**Figure 1:**
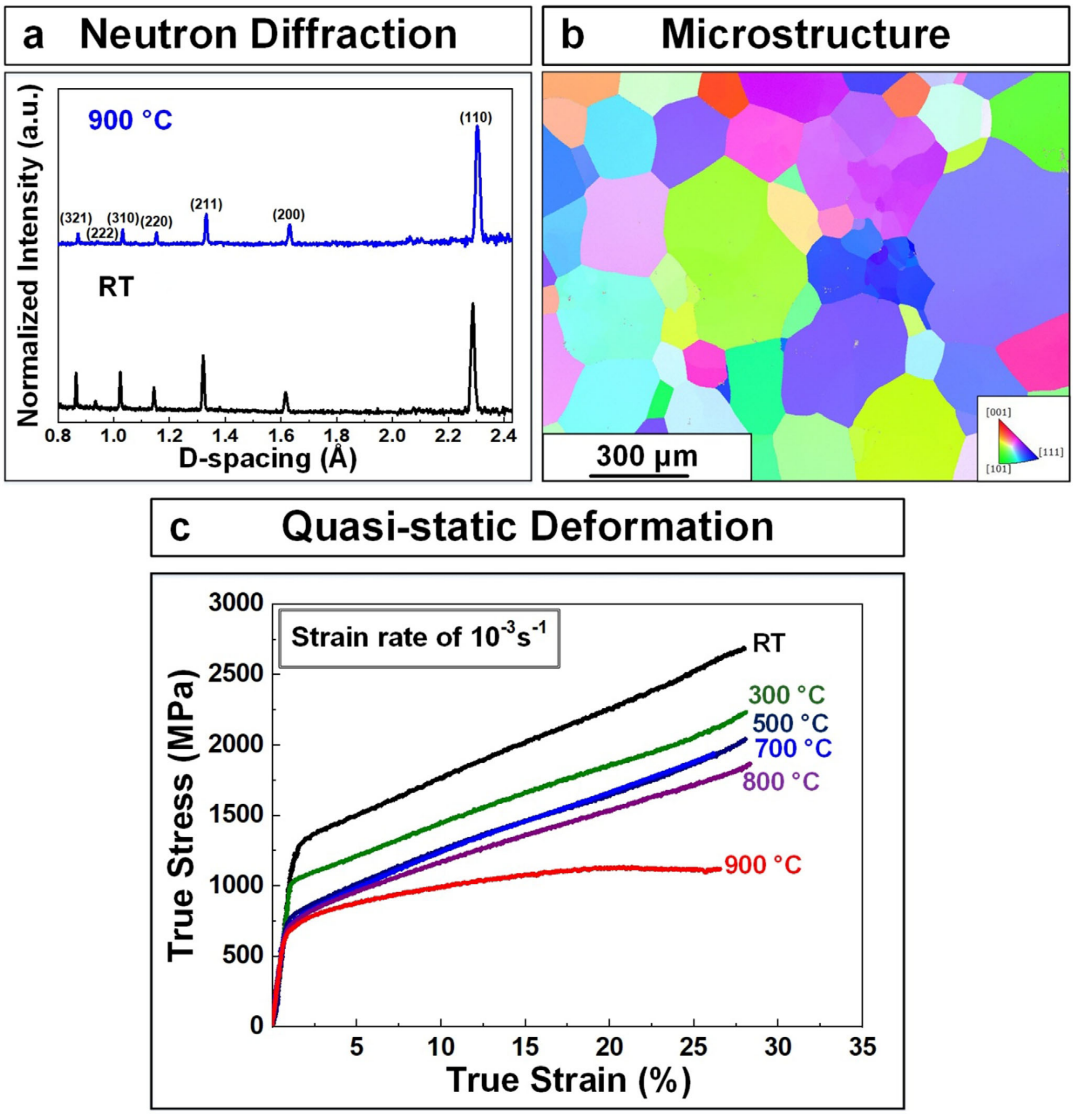
Phase, microstructure, and mechanical properties of NbTaTiV RMPEA. a) Neutron‐diffraction patterns of the homogenization‐treated NbTaTiV RMPEA at RT and 900 °C. b) EBSD‐IPF image. c) Compressive true SS curves obtained at RT and elevated temperatures with strain‐rate of 10^−3^ s^−1^.

**Figure** **1**a shows the neutron diffraction patterns for the heat‐treated NbTaTiV RMPEA at RT and 900 °C. The major diffraction peaks are identified as corresponding to the formation of single BCC‐phase with lattice constants of 3.232 Å (RT) and 3.259 Å (900 °C), respectively, showing excellent phase stability at high temperatures. The slight change in the lattice constant can be attributed to the thermal expansion of the material at 900 °C. The EBSD analysis of this material also showed the presence of randomly oriented equiaxed grains with a relatively large grain size (200 to 400 µm), as shown by IPF in Figure 1b.

### 2.2 Mechanical Behavior of NbTaTiV RMPEAs as a Function of Strain‐Rate and Temperature

**Figure 2:**
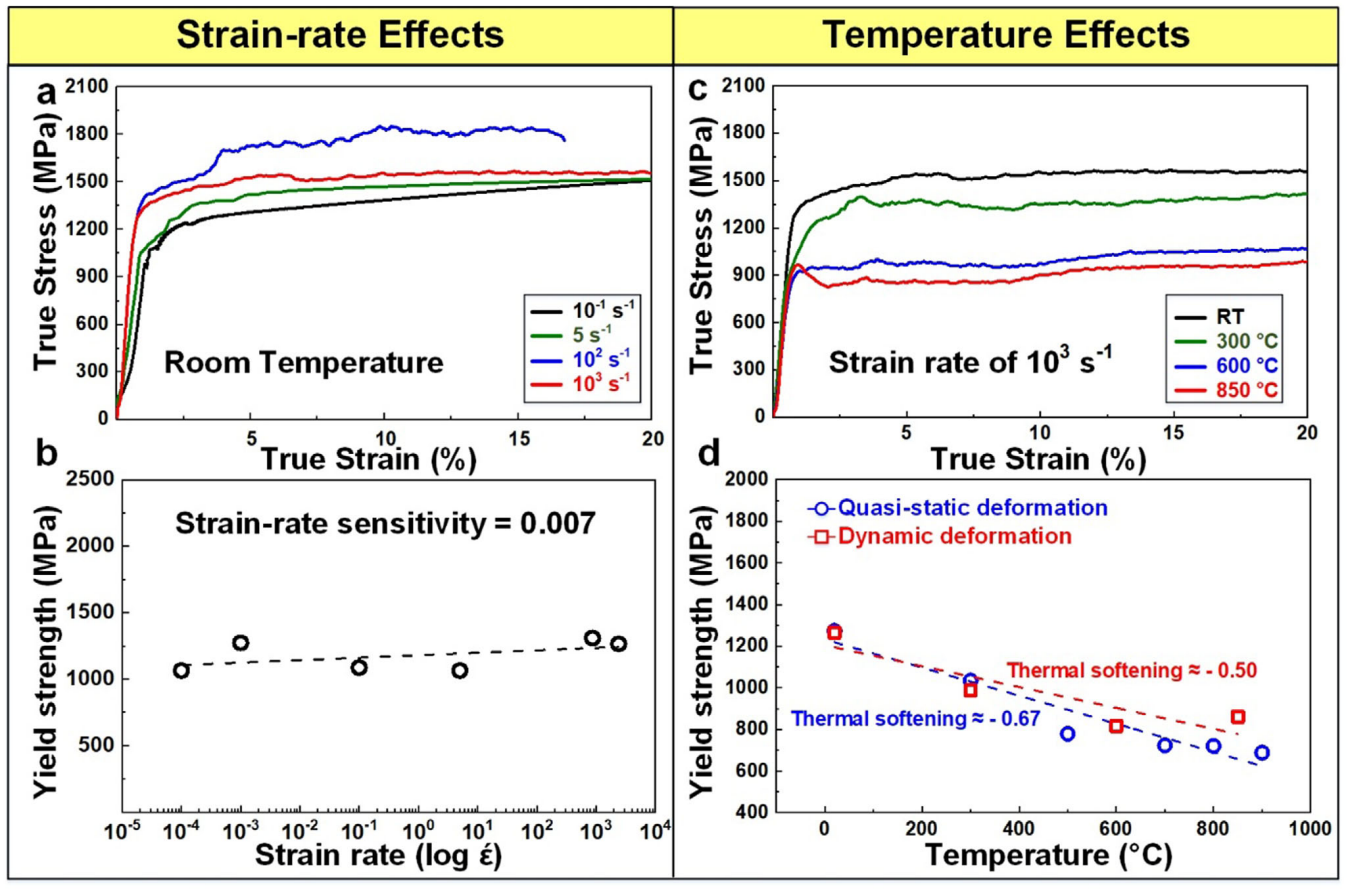
Mechanical behaviors as a function of strain rate and temperature. a) True SS curves of the NbTaTiV RMPEA at various strain rates. b) Evolution of yield stress as a function of logarithm strain rate. c) True SS curves obtained at room and elevated temperatures with a strain rate of 10^3^ s^−1^. d) Evolution of yield strength as a function of temperature at quasi‐static and dynamic loading.

Figure 1c shows true stress–strain (S–S) curves for the homogenization‐treated NbTaTiV under the uniaxial compression obtained at temperatures ranging from RT to 900 °C at a strain rate of 1 × 10^−3^ s^−1^. **Table** **1** details the yield strength values of NbTaTiV as a function of temperature and strain rate. The present NbTaTiV alloy has a high yield strength at RT (1,273 MPa) and significant strain‐hardening fracture occurring at a compressive strain limit of 30%. As the temperature increases, the yield strength gradually decreases to 688 MPa (900 °C) but strain hardening is still observed up to 800 °C, implying excellent resistance to softening at high temperatures. As the strain rate increases to 1 × 10^−1^ and 2 × 10^3^ s^−1^, the S–S curves shown in **Figure** **2**a exhibit a slightly enhanced yield strength from 1085 (1 × 10^−1^ s^−1^) to 1,264 MPa (2 × 10^3^ s^−1^) with an absence of strain‐hardening at high strain‐rates, compared with the quasi‐static data (Figure 1c). The strain‐rate effect on yield strength is quantitatively analyzed via a strain‐rate sensitivity parameter, defined by the slope of the logarithmic yield strength versus strain rate ($\frac{\log\sigma_{y}}{\log\dot{\varepsilon }})$, as presented in Figure 2b. The corresponding strain‐rate sensitivity of NbTaTiV is ≈0.007, exhibiting a low strain‐rate dependence of the yield strength. To study the effect of temperature on yield strength at high strain rates, compression tests were conducted at elevated temperatures at a strain rate of 2500 s^−1^ (i.e., 10^3^ s^−1^), as indicated in Figure 2c. Similar to the quasi‐static data, the yield strength of the NbTaTiV tested under dynamic loading gradually decreased from 1265 (RT) to 860 MPa (850 °C). This decrease in yield strength is quantified in Figure 2d and compared to data from quasi‐static tests. Specifically, the thermal softening behavior in the NbTaTiV RMPEA is quantified via the slope of the yield strength with temperature ($\frac{d\sigma_{y}}{dT}$). The obtained thermal softening values at quasi‐static and dynamic deformation are − 0.67 and − 0.50, respectively, indicating that the NbTaTiV RMPEA maintains high strength at high temperatures irrespective of strain rates. Strain‐rate insensitivity of the present alloy at high temperatures is critical in case of its consideration as an alloy to be used in high‐temperature applications.

**Table 1:** The detailed values of yield strengths as a function of strain‐rates and temperature.

Parameters	Yield strength quasi‐static loading (10^−3^ s^−1^)	Parameters	Yield strength dynamic loading (10^3^ s^−1^)
temperatures		temperatures	
−187 °C	963 MPa	RT	1265 MPa
RT	1273 MPa	300 °C	987 MPa
300 °C	1034 MPa		
500 °C	778 MPa	600 °C	816 MPa
700 °C	723 MPa		
800 °C	720 MPa	850 °C	860 MPa
900 °C	688 MPa		

**Figure 3:**
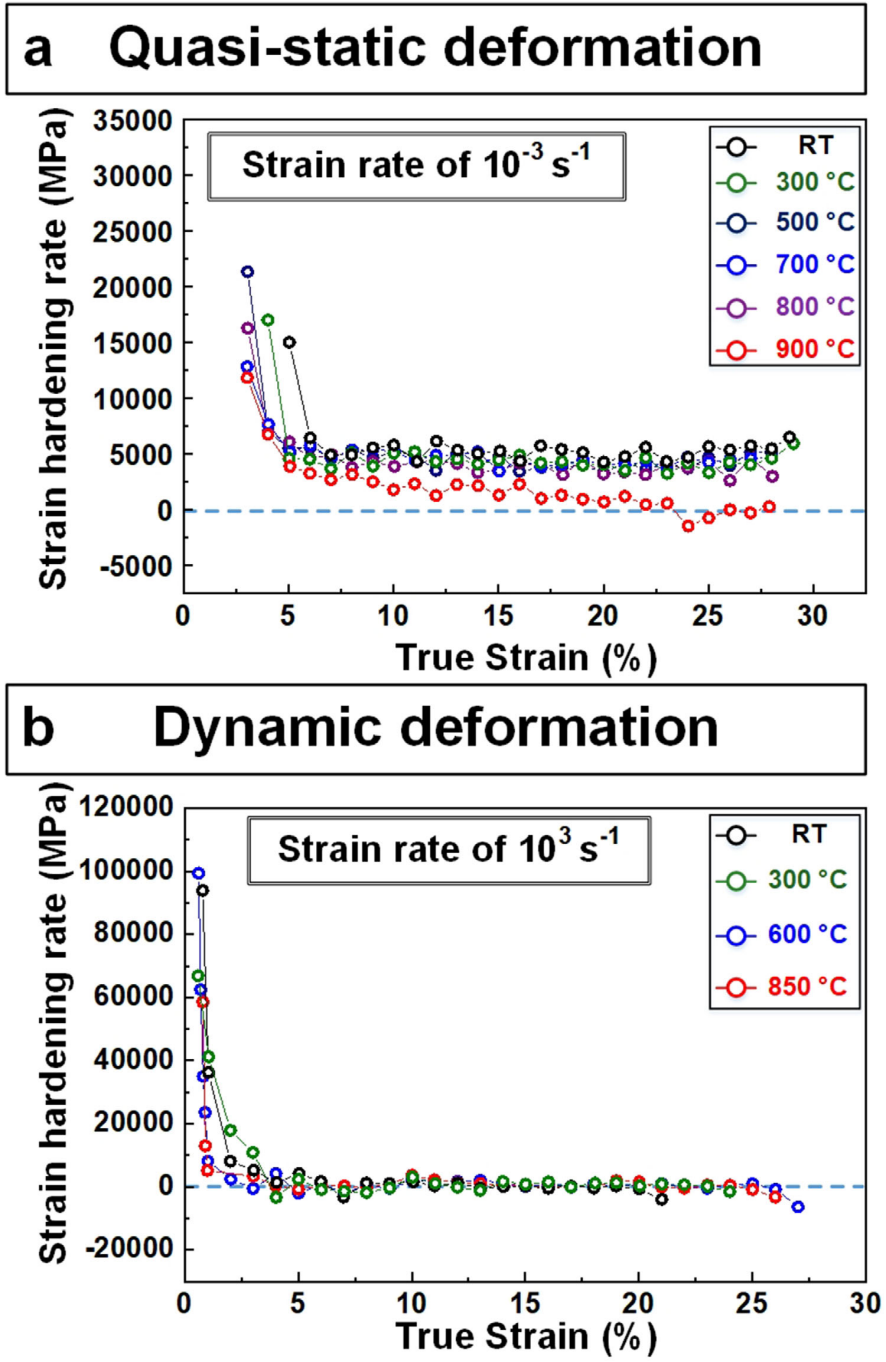
Strain‐hardening rate as a function of true strain for NbTaTiV at room and elevated temperatures during quasi‐static and dynamic deformation. a) Under quasi‐static conditions (strain‐rate of 10^−3^ s^−1^), strain‐hardening behavior is observed from room temperature to 900 °C. b) Strain‐hardening is observed under dynamic deformation (strain‐rate of 2500 s^−1^, i.e., 10^3^ s^−1^) from room temperature to 850 °C.

**Figure** **3**a,b shows the evolution of the strain‐hardening rate, which is defined by ($\frac{d\sigma }{d\varepsilon }$) in the plastic regime as a function of true strain during quasi‐static (10^−3^ s^−1^) and high (10^3^ s^−1^) strain‐rate loading at elevated temperatures. After elastic deformation, the strain‐hardening rate reaches a steady state during quasi‐static deformation from RT to 800 °C (Figure 3a). The values of steady‐state strain‐hardening rates are ≈3100 to 5400 MPa. As the temperature increases up to 900 °C, the strain‐hardening rate gradually decreases and reaches a negative value after 23% plastic deformation, which is associated with thermal softening at 900 °C. On the other hand, the strain‐hardening rate during dynamic deformation (10^3^ s^−1^) was measured to be negligible at all temperatures, as presented in Figure 3b, showing the absence of strain‐hardening/softening during plastic deformation. In general, data associated with strain‐hardening rates is related to specific deformation mechanisms.

For typical BCC‐phase metals and alloys, an increase in the hardening rate mainly originates from the formation of mechanical‐deformation twins,^[^ ^36^, ^37^ ^]^ which can disrupt dislocation motion, leading to an increase in the strain‐hardening rate. In contrast, a gradual reduction in the strain‐hardening rate arises from uninterrupted dislocation motion and/or a high sensitivity to adiabatic heating causing rapid thermal softening leading to easier dislocation motion.^[^ ^30^ ^]^

### 2.3 Microstructural Evolution during Deformation at High Strain Rates

**Figure 4:**
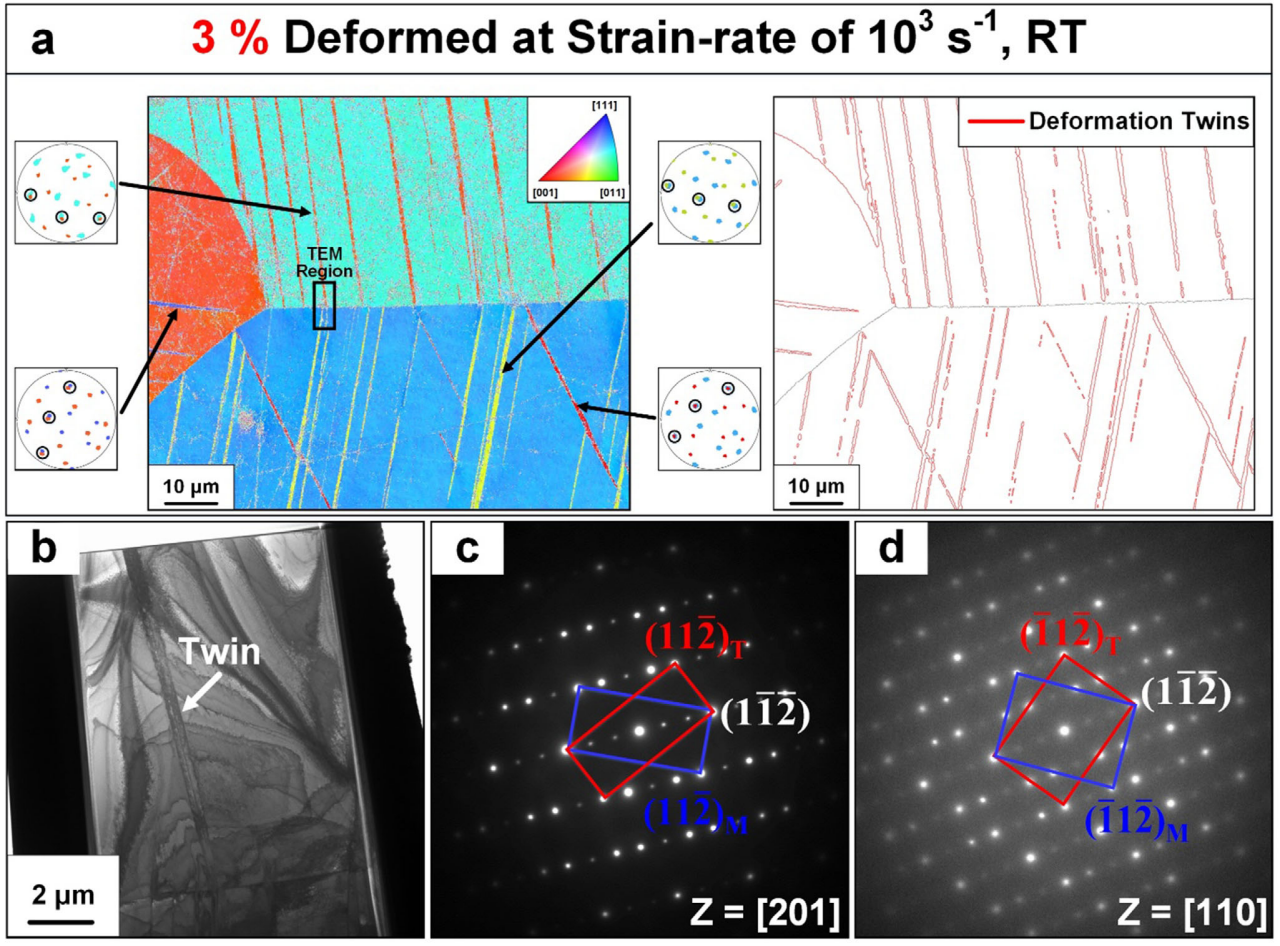
Microstructure of a 3%‐deformed NbTaTiV RMPEA at a strain rate of 10^3^ s^−1^. a) IPF color map and deformation twin with the axis of the sample‐surface‐normal direction collected from the 3%‐deformed NbTaTiV RMPEA at a strain‐rate of 10^3^ s^−1^. The pole figure of {112} planes of grains in circled regions is presented. b) TEM bright‐field image of the specimen extracted from the black boxed area in (a). c,d) selected area electron diffraction (SAED) patterns with beam directions of c) [201] and d) [110].

To further understand the data obtained at high strain rates, the microstructure of the NbTaTiV was examined, using EBSD‐IPF post‐testing at elevated temperatures (from RT to 850 °C), as shown in Figure S1 (Supporting Information). A large density of features resembling “slip bands” is distributed within the interiors of different grains in deformed samples at RT (Figure S1a, Supporting Information). Furthermore, it is clear from the IPFs that there is no overall change in the texture of the materials upon testing at RT in comparison to the undeformed sample (Figure 1b). As the testing temperature increases from 300 °C to 850 °C, the density of slip bands decreases, as compared to RT (Figure S1b–d, Supporting Information). To determine the exact nature of the “thin” bands in the interior of the grains, high‐magnification EBSD micrographs were collected from a sample that was deformed up to a 3% strain at RT with a strain rate of 10^3^ s^−1^. **Figure** **4**a reveals the IPF color map where multiple thin lamella‐type deformation bands are formed in the matrix. In the IPF map, the color of these deformation bands is different from that of the matrix, which indicates that they have distinct orientations. The pole figures of the planes in the matrix and the deformation bands are illustrated on the side of the figure, and the shared planes for corresponding regions are indicated. All these shared planes belong to the same family plane, which implies that these deformation bands can be twins. The average thickness of the twin, measured from the right side of Figure 4a, is about 0.43  µm. To further verify this assertion, a planar‐view TEM specimen was extracted from the black box in Figure 4a. The bright‐field (BF) TEM image of this specimen is shown in Figure 4b. Diffraction patterns were obtained from the boundary between the deformation bands and the matrix. Figure 4c,d shows diffraction along the [201] and [110] zone axis. The indexed patterns show mirror symmetry across the (1$\bar{1}\bar{2}$) plane or 180° rotation around the normal of the (1$\bar{1}\bar{2}$) plane, highlighting the formation of type‐I twins during high strain‐rate deformation at RT.

This result confirms that not only the formation of twins during plastic deformation at high strain rates, but it is also worth noticing that the twins are much thinner than typical deformation twins.^[^ ^38^ ^]^ Note that the average twin thickness of 0.43  µm in NbTaTiV, which is thinner in comparison to conventional 𝛽‐Ti alloys (2–3 µm).^[^ ^39^ ^]^ It is further found that the thickness of these twins tends to decrease as they approach the grain boundaries. This variation of the twin thickness is shown in Figure 4b. In fact, deformation twins often have a lenticular shape and are thicker at grain boundaries since deformation twinning is formed by the nucleation, propagation, and thickening processes.^[^ ^40^ ^]^ During nucleation under the influence of strain energy and thermal fluctuations, twin embryos usually nucleate at grain boundaries. Then, the twins thicken and propagate by the nucleation and expansion of twinning disconnections. During the thickening process, more and more twinning disconnections are generated, and form steps or facets on the twin boundaries, which results in the formation of lenticular‐shape twins. In the present case, based on the shape of the thin twins, there is an indication of the absence of significant thickening, which suggests that there must be a different source of twinning disconnections. Moreover, EBSD image (in Figure 4a) suggests that the twins are nucleating within grains rather than at grain boundaries. Based on this analysis, we hypothesize that the high strain rate provides extremely high energy to the alloy, which exceeds the criteria for intragranular twin nucleation but limited lateral growth of the twins. The high oblateness of the twins (length / width) suggests a high twinning shear stress for nucleating the twins, and the lenticular shape is consistent with the theory that the twin shape is governed by the back stress produced by the twinning shear.^[^ ^41^, ^42^, ^43^ ^]^ The twin embryos with limited thickness are postulated to form at multiple areas inside the grains. For each twin embryo, a limited number of twinning disconnections glide and accommodate deformation. The microstructural evolution as a function of plastic strain (3, 6, and 20%) at a strain‐rate of 10^3^ s^−1^ are included in Figure S2 (Supporting Information), indicating that there is no distinct increase in the thickness of twins with increase of plastic strain at RT.

### 2.4 Comparison of the Temperature and Strain‐Rate Dependence of Yield Strength

**Figure 5:**
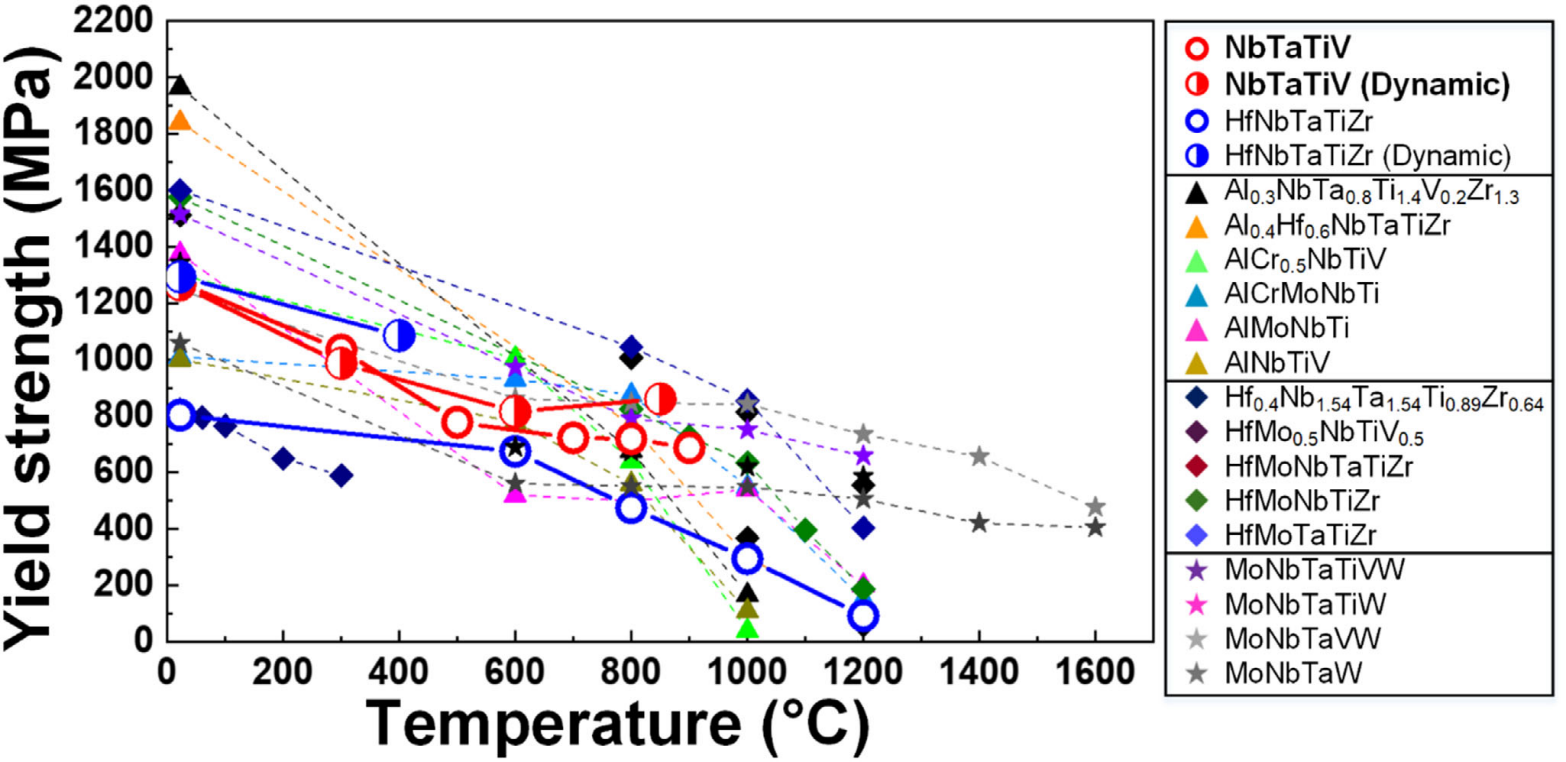
Comparison of the temperature dependence of yield strength for the NbTaTiV RMPEA (present work) with various existing single BCC‐phase MPEAs. The yield strength versus temperature curves at dynamic deformation for NbTaTiV and HfNbTaTiZr are included.^[^ ^89^, ^90^ ^]^

The yield strength as a function of temperature in the present NbTaTiV RMPEA is compared with previously reported MPEAs at quasi‐static rates (10^−3^ s^−1^), as shown in **Figure** **5**. Note that all listed MPEAs are single BCC‐phase structures at room as well as high temperatures. The evolution of yield strength at a high strain rate (10^3^ s^−1^) for the NbTaTiV as compared to the reported HfNbTaTiZr RMPEA is also included (half‐filled symbol).^[^ ^29^ ^]^ Although Al‐ (Al_0.3_NbTa_0.8_Ti_1.4_V_0.2_Zr_1.3_ and Al_0.4_Hf_0.6_NbTaTiZr) and Hf‐containing (HfMoNbTiZr, HfMoNbTaTiZr, and HfMoTaTiZr) MPEAs possess higher yield strength than the present NbTaTiV at RT, their yield strength dramatically decreases as a function of temperature.^[^ ^44^, ^45^, ^46^, ^47^, ^48^ ^]^ In contrast, Mo‐containing RMPEAs (MoNbTaW, MoNbTaTiW, MoNbTaVW, and MoNbTaTiVW) and the present NbTaTiV show a lower reduction in yield strength at elevated temperatures.^[^ ^15^, ^49^ ^]^ Moreover, the yield strength of these materials is higher than other reported MPEAs above 800 °C. The RT yield strength of the HfNbTaTiZr RMPEA at high strain rates is 1.6 times higher than its quasi‐static counterpart.^[^ ^29^ ^]^ In contrast, the yield strength of the present NbTaTiV remains relatively constant at high strain rates and elevated temperatures. Figure S3 (Supporting Information) compares the strain‐rate sensitivity of pure aluminum as well as FCC‐ and BCC‐phase MPEAs, demonstrating that NbTaTiV RMPEA exhibits notably low strain‐rate sensitivity (0.007). It also exhibits less strain‐rate dependence on yield strength in a wide temperature range. In fact, the dominant strengthening mechanism in NbTaTiV is solid‐solution strengthening, which is mainly caused by severe lattice distortion. The distorted lattice could effectively reduce the diffusivity of elements at high temperatures, leading to enhanced resistance to thermal softening during both quasi‐static and dynamic loading.

## 3 Discussion

### 3.1 Effect of Strain Rate on Yield Strength

In general, the yielding and strain‐hardening behavior in metallic materials is substantially influenced by strain rates.^[^ ^37^, ^50^, ^51^, ^52^ ^]^ High strain‐rate loading is well known to form dislocation cells that are smaller in size for both FCC and BCC‐phase pure metals, which usually contributes to a uniform distribution of dislocations.^[^ ^51^, ^53^, ^54^ ^]^ Hence, more dislocations tend to be trapped within cell interiors.^[^ ^55^ ^]^ When dislocations activate through the crystal lattice, the long and short‐range barriers prevent the movement of dislocations.^[^ ^56^, ^57^ ^]^ The short‐range barrier, such as the Peierls potential, can be surmounted by the thermal‐activation energy under a quasi‐static loading condition.^[^ ^58^ ^]^ However, the fast deformation process under a dynamic‐loading condition lacks sufficient time to overcome the thermal barrier, resulting in greater difficulty for dislocation motion, consequently, it leads to reduced dislocation tangling, dislocation line length storage, and lower rate of strain hardening.^[^ ^59^, ^60^, ^61^ ^]^ Thus, higher stresses are required to promote dislocation motion, which strongly contributes to high yield strength at higher strain rates in contrast to quasi‐static deformation.

In the case of MPEAs, the intrinsic chemical complexity leads to notable solid‐solution strengthening effects due to the presence of many solute atoms with varying atomic sizes.^[^ ^4^, ^35^ ^]^ These atomic size mismatches induce severe lattice distortion and generate long‐range lattice friction, which significantly reduces dislocation mobility. In particular, in some BCC‐phase RMPEAs, the randomly distributed solutes introduce large intrinsic energy barriers that inhibit the glide of thermally activated edge dislocations.^[^ ^32^, ^62^ ^]^ These unique strengthening mechanisms, including the interaction between edge dislocation and a distorted lattice environment, could lead to high yield strength regardless of the imposed strain rate.

Recent studies by Dirras et al.,^[^ ^28^ ^]^ Hu et al.,^[^ ^29^ ^]^ and Zhang et al.,^[^ ^30^ ^]^ investigated the strain‐rate‐dependent deformation behavior of single BCC phase HfNbTaTiZr and HfNbTaTiZrMo RMPEAs, employing compression tests with varying strain‐rates from 10^−4^ to 10^3^ s^−1^. Compression tests over a wide range of strain rates revealed a pronounced increase in yield strength with strain rate, indicating a strong positive strain‐rate sensitivity. This behavior was attributed to the dominance of screw dislocation motion,^[^ ^63^, ^64^ ^]^ which is still contributed by a relatively high Peierls barrier and similar to conventional BCC metals and alloys. Note that strain‐rate sensitivity of HfNbTaTiZrMox (*x* = 0.25, 0.5 and 0.75) indicate strain‐rate sensitivity in the range of 0.016 to 0.021 in comparison to 0.007 observed in the present NbTaTiV, as shown in the Figure S3 (Supporting Information).

In contrast, the present NbTaTiV alloy exhibits excellent mechanical stability at various strain rates, with markedly lower strain‐rate sensitivity (Figure 2b). This suggests a unique strengthening mechanism, which is governed by edge dislocation interactions with severe lattice distortion, rather than resistance of screw dislocation motion. Strengthening in present NbTaTiV RMPEA has been previously studied using integrated experimental and theoretical approaches, which collectively indicate that solid‐solution strengthening arising from lattice distortion is the dominant mechanism.^[^ ^3^, ^32^ ^]^

Two distinct mechanisms are generally considered responsible for high strain‐rate strengthening in BCC‐phase alloys: (1) hindered screw dislocation motion due to high Peierls barriers and reduced dislocation cell size, as in conventional BCC alloys,^[^ ^65^ ^]^ and (2) restricted edge dislocation glide due to severe lattice distortion, which is more prominent in some RMPEAs. In the NbTaTiV alloy, severe lattice distortion could create intrinsic energy barriers that obstruct edge dislocation glide, even under quasi‐static loading. In fact, the nucleation and movement of dislocations during elastic deformation and onset of yielding in BCC‐phase alloys varied in different grains due to their orientation‐dependent slip, Peierls potential, and Schmid factors.^[^ ^66^ ^]^ However, the severely distorted lattices in the NbTaTiV result in the orientation‐independent elastic deformation and yielding behaviors (i.e., elastically isotropic deformation) during quasi‐static deformations.^[^ ^33^, ^62^ ^]^ Especially, small variations of grain‐to‐grain yielding sequences in differently oriented grains could lead to the resistance of plastic deformation with high yield strength even under quasi‐static loading conditions. In general, the evolution of lattice strains is dependent on grain orientations during elastic deformation due to varying atomic‐bond strengths, stiffnesses, and their interaction with dislocations.^[^ ^67^ ^]^ These large variations of grain‐to‐grain yield sequences could reduce the achievable yield strength of BCC‐phase materials.^[^ ^66^, ^68^ ^]^ While a modest increase in yield strength is observed with increasing strain rate (Figure 2a), the substantial energy barriers for edge dislocation movement, introduced by the lattice distortion, sustain high strength under both quasi‐static and dynamic loading conditions. To provide a rational explanation for this low strain‐rate sensitivity behavior, and to distinguish it from screw‐dominated strengthening seen in other RMPEAs, it is essential to verify whether the dominant dislocation mechanism in NbTaTiV is indeed governed by edge dislocations. Therefore, the dominant dislocation character in the NbTaTiV RMPEA is experimentally identified in the following section.

### 3.2 Identification of Dominant Dislocation Types for the NbTaTiV Alloy at Elevated Temperatures

BCC‐phase metallic materials commonly exhibit different mobility of dislocations between edge and screw, indicating faster mobility in edge character, compared to screw dislocations due to their core properties during plastic deformation.^[^ ^68^ ^]^ The critical stress required to induce the movement of edge dislocation is notably small (< 6 MPa for the pure BCC‐phase Nb), resulting in the propensity for edge dislocation annihilation.^[^ ^69^ ^]^ Consequently, it results in the screw/edge anisotropy in the apparent mobility deviation.^[^ ^70^ ^]^ Therefore, the remaining dislocations in BCC‐phase metals and alloys tend to have more screw character.

However, Rao et al. have reported atomistic simulations of plastic‐deformation behavior for the BCC‐phase MPEAs (CoFeNiTi) in terms of dislocation motion.^[^ ^71^ ^]^ This in‐depth investigation predicts the tendency of dislocation motions in BCC‐phase solid‐solution MPEA to correspond to the random elemental distribution, which is considerably different from conventional BCC‐phase metals and alloys. During the plastic deformation of CoFeNiTi, the edge dislocation core can deviate from their neutral dislocation planes, producing wavy dislocation lines, which are attributed to the severe lattice distortion. This feature is induced by compositional fluctuations and, thus, leads to notable difficulty in the edge dislocation movement in addition to acting as a strengthening mechanism. A similar trend of dislocation mobility was reported by Xu et al. in MoNbTi and NbTiZr RMPEAs, exhibiting reduced anisotropic mobility of edge and screw dislocations, which contrasts with the conventional BCC‐phase metals and alloys.^[^ ^72^ ^]^

**Figure 6:**
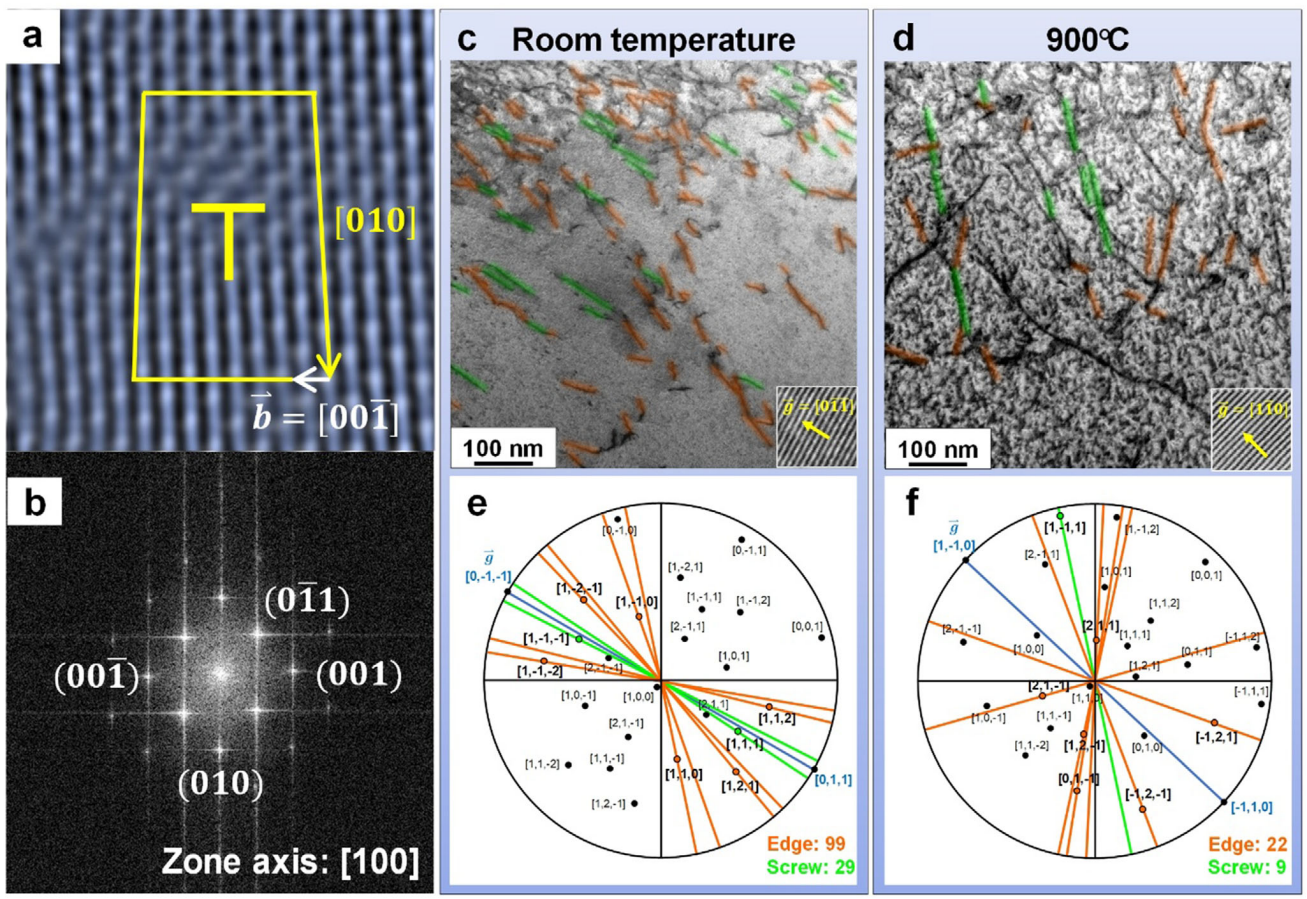
STEM‐HAADF and STEM‐ABF images showing the dominance of edge dislocations in NbTaTiV at RT and 900° C. a) The filtered atomic‐resolution HAADF image of a 11.8%‐deformed NbTaTiV RMPEA projected along the [100] direction as shown in the b) corresponding Fast Fourier Transform (FFT) spectrum. The STEM‐ABF image of the 11.8% strained NbTaTiV at c) RT and d) 900 °C with a two‐beam condition near the [100] zone axis. The inset is the HAADF image, indicating that the direction of the $\vec{g}=\left(0\bar{1}\bar{1}\right)$ for RT and $\vec{g}=\left(1\bar{1}0\right)$ for 900 °C. Stereographic projection related to the [110] orientation, where e) $\left(0\bar{1}\bar{1}\right)$ for RT and f) $\left(1\bar{1}0\right)$ for 900 °C have been aligned with images (c,d). All possible dislocations are indicated, and those corresponding to the images in (c,d) are highlighted in bold. The orange and green lines represent edge and screw dislocations, respectively. As a result, ≈77% of the dislocations are identified as edge at RT and ≈71% of the dislocations are identified as edge at 900 °C.

Based on the reported prediction of plastic deformation behaviors with edge dislocations in BCC‐phase MPEAs,^[^ ^32^ ^]^ the types of dislocations in the present NbTaTiV alloy, which has severely distorted lattices, are investigated by a high‐angle annular dark‐field imaging‐scanning transmission electron microscope (HAADF‐STEM) and annular bright field (ABF) images coupled with stereographic, as shown in **Figure** **6**. Figure 6a,b presents the filtered atomic‐resolution HAADF image of 11.8%‐deformed NbTaTiV RMPEA projected along the [100] direction as shown in the corresponding fast Fourier transform (FFT) spectrum. In Figure 6a, the yellow marks indicate the edge dislocation and Burgers circuit, and the white arrow is the Burgers vector, $\overset{⇀}{b}=\;\left[00\bar{1}\right]$. Figure 6c,d show the STEM‐ABF images of dislocation networks in a 11.8%‐deformed NbTaTiV at RT and 900 °C, which is taken near the [100] zone axis. All dislocations having a line of length over 5 nm are identified as orange (edge) and green (screw) lines. The inset is the HAADF image, indicating the direction of $\vec{g}=\left(0\bar{1}\bar{1}\right)$ for RT and $\vec{g}=\left(1\bar{1}0\right)$ for 900 °C. Figure 6e,f present the results of stereographic‐projection analysis, which exhibits the characters of these long straight dislocation lines. The observed dislocations are highlighted in bold and lie along the lines presented. It was obtained that the measured dislocations are predominantly of edge character at RT (99 out of 128) and maintained edge‐dislocation‐dominant deformation at 900 °C (22 out of 31).

### 3.3 Effect of Strain‐Rate on Strain‐Hardening

The present NbTaTiV RMPEA exhibits negligible strain hardening during dynamic deformation at elevated temperatures (Figure 2c,d). In general, strain‐hardening in metallic materials is sensitive to both strain rate and temperature, but the underlying mechanisms differ by crystal structure. In FCC‐phase alloys, especially those with low stacking fault energy (SFE), deformation twinning and suppression of dynamic recovery contribute to enhanced strain hardening under high strain‐rate loading.^[^ ^65^, ^73^ ^]^ In contrast, BCC and HCP‐phase metals, which typically have higher SFE and lattice friction, exhibit strain hardening that is less sensitive to strain rate and temperature after yielding.^[^ ^50^, ^57^ ^]^

In single FCC‐phase MPEAs, the fundamental strain‐hardening mechanism is similar to conventional FCC‐phase materials, as discussed above. For example, the Al_0.3_CoCrFeNi MPEA, which possesses a low SFE (below 30 mJ m^−2^), shows high strain‐hardening during both quasi‐static and high strain‐rate loading.^[^ ^23^ ^]^ The slopes of strain‐hardening rates changed from negative to positive during plastic deformation at a strain rate of 1 × 10^3^ s^−1^, indicating a transition in the deformation mode from dislocation slip to deformation twinning. Mishra et al. further suggested that high lattice strains in FCC‐phase MPEAs could play a critical role in strain‐hardening in contrast to conventional FCC‐phase alloys.^[^ ^74^ ^]^ The lattice strains in MPEAs increase the intrinsic energy of the crystal, which results in the reduction of the required energy for the nucleation of dislocations and twins under high strain‐rate deformation. Therefore, the dislocation‐slip and interaction between twins can lead to a higher strain‐hardening rate and resistance to shear localization.^[^ ^23^ ^]^

In contrast, strain‐hardening in single BCC‐phase RMPEAs during high strain‐rate loading is different from other conventional BCC‐phase materials. Due to the high lattice friction in BCC‐phase MPEAs, the movement of dislocations could be effectively hindered in the distorted lattice.^[^ ^4^ ^]^ This would usually lead to an increase in the strain‐hardening rate during high strain‐rate loading. However, this mechanism competes with the thermal‐softening behavior due to adiabatic heating. It is a well‐known fact that thermal softening, which is mainly attributed to adiabatic heating during dynamic loading, can strongly influence strain‐hardening behavior.^[^ ^12^ ^]^ In fact, metallic alloys often produce significant heat during plastic deformation, and the heat‐generation rate exceeds that of the heat loss at high strain rates, which leads to a dramatic increase in temperature within a short time.^[^ ^75^, ^76^ ^]^ Hence, the two competing mechanisms above (strain‐hardening by dislocation motions and softening by adiabatic heating) could dominate during plastic deformation at high strain rates.

### 3.4 Deformation Behavior at Cryogenic Temperatures

**Figure 7:**
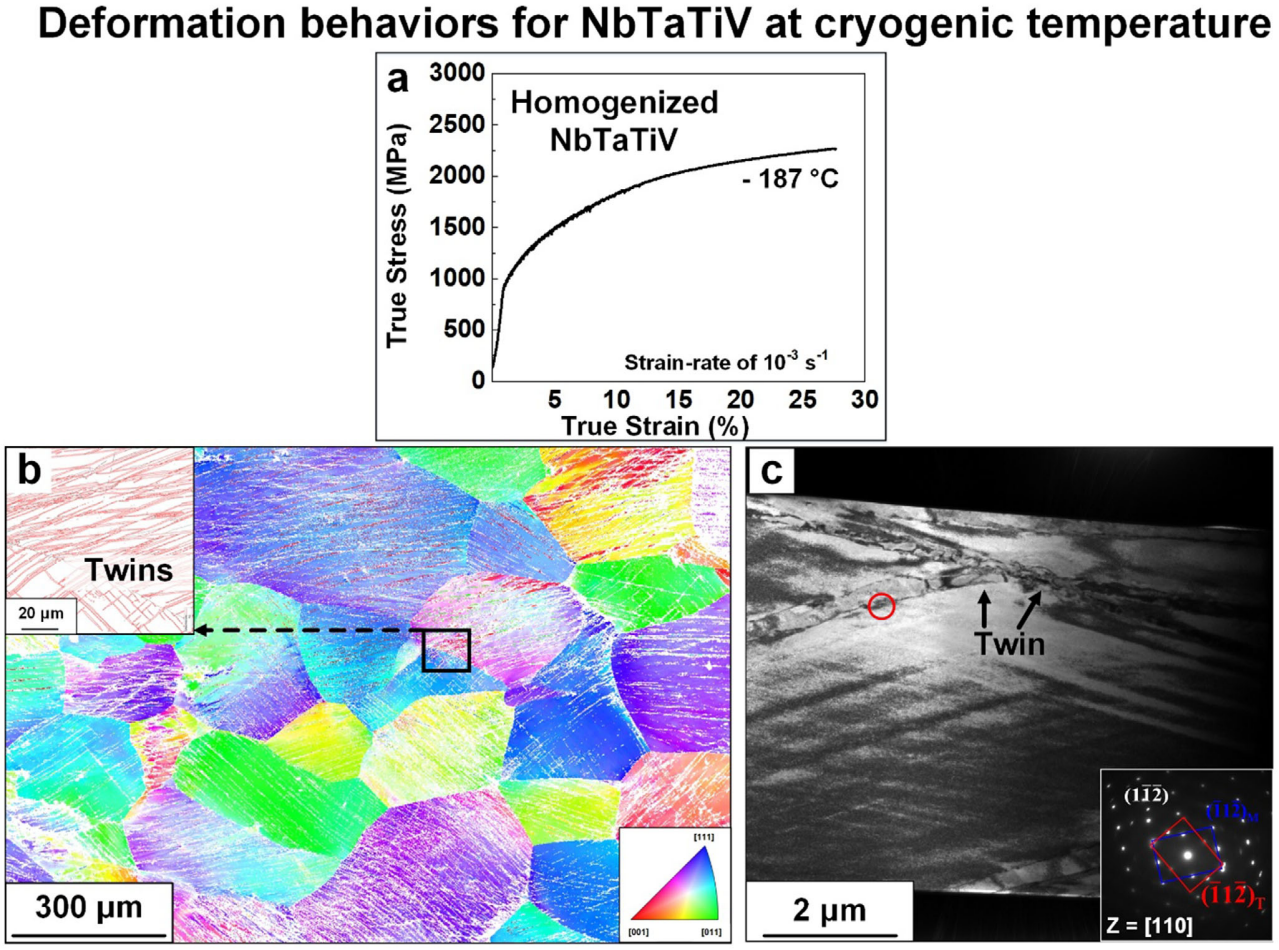
Deformation behavior for the NbTaTiV RMPEA and microstructural evolution after deformation at cryogenic temperature. a) Compressive true SS curves obtained at ‐187 °C with a strain‐rate of 10^−3^ s^−1^. b) IPF color map with the axis of a sample‐surface‐normal direction collected from the 10%‐deformed NbTaTiV RMPEA at a cryogenic temperature (‐187 °C). c) TEM bright‐field image of cryogenic‐temperature‐deformed NbTaTiV and selected area electron diffraction patterns with a beam direction of [110] zone axis.

Typically, the deformation behavior in BCC‐phase materials under high strain rates is similar to that observed at low temperatures under quasi‐static loading.^[^ ^65^ ^]^ For example, the size of dislocation cells in pure niobium under high strain rate and low‐temperature conditions are small, promoting a more uniform dislocation distribution with an increase dislocation density.^[^ ^53^, ^77^ ^]^ The trend of uniform dislocation networks and suppression of the cell formation further leads to a reduction in cross‐slip.^[^ ^78^ ^]^ In BCC‐phase metals, reduced cross‐slipping at low temperatures could be associated with the high Peierls barrier. In order to further understand the deformation behavior at low temperatures in NbTaTiV, cryogenic compressive tests (– 187 °C) at a strain rate of 10^−3^ s^−1^ were performed, coupled with TEM on the post‐mortem samples, as presented in **Figure** **7**. The yield strength of NbTaTiV at ‐187 °C is about 963 MPa with substantial strain‐hardening (Figure 7a). The EBSD map was collected from the deformed sample to investigate the reason behind the substantial strain hardening, as presented in Figure 7b. The EBSD image clearly shows the formation of many twins and active twin‐twin interaction sites. A piece of the sample was thereafter extracted from the magnified region using a focused ion beam (FIB) and prepared for the TEM analysis. Figure 7c shows the BF‐TEM micrograph of the 10%‐deformed sample. Two twins intersecting with each other are seen in the top part of this figure. Unlike typical deformation twins with lenticular shapes, these twins exhibit irregular morphologies with non‐planar boundaries and large steps along with twin planes. Diffraction patterns were collected in the red circled area and added as an inset in Figure 7c. The twin orientation was thereby verified. The viewing direction of the diffraction pattern is [110]. Notably, the spots associated with the (1$\bar{2}\bar{2}$) plane in the twin and matrix respectively do not perfectly overlap. This irregularity is related to the accumulation of twinning dislocations at the stepped interfaces. A similar phenomenon was detected near the tip of other deformation twins. These microstructural characteristics could suggest two potential mechanisms for the enhanced strain hardening at low temperatures. First, twin‐twin interactions may contribute to increased strain‐hardening. Second, the presence of large steps along the twin boundaries implies that twinning involves multiple nucleation events. The low temperature impedes the glide of the twinning dislocations, and more nucleation is activated under loading, promoting repeated nucleation. Initially, nucleation occurs at grain boundaries, but with increasing strain, less favorable sites within grain interiors are activated. This transition requires additional energy, resulting in increased flow stress with significant strain hardening. Note that due to current experimental limitations, specifically, the lack of a liquid nitrogen‐compatible setup for high strain‐rate testing, dynamic deformation at cryogenic temperatures could not be investigated in this study. However, further studies exploring dynamic strain‐rate effects at cryogenic temperatures are essential to establish a more comprehensive understanding of the deformation response of RMPEAs under extreme environments. This will be proposed as future work once the cryogenic testing capability is established.

### 3.5 Estimation of Stacking Fault Energy and Twinning‐Nucleation Stress

**Figure 8:**
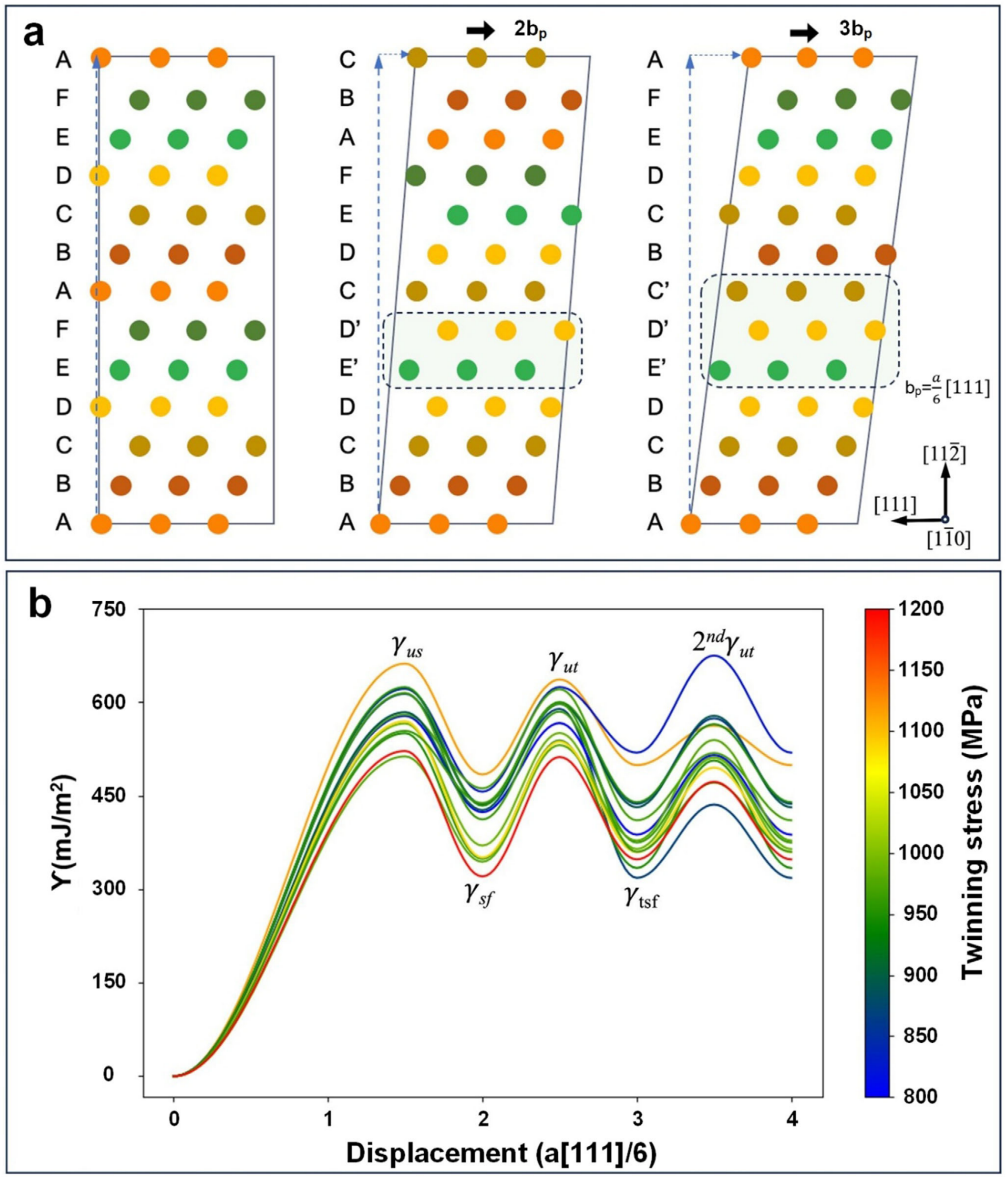
Atomistic illustration of stacking sequence in a BCC lattice and the GPFE of the NbTaTiV RMPEA. a) Perfect BCC lattice, the two‐layer stacking fault structure is formed by applying 2 *b*_p_ displacements, and three‐layer twinning fault structure is developed by applying 3 *b*_p_ displacements. b) 15 different configurations’ GPFE curves, which indicates γ_us_, γ_sf_,γ_ut_,γ_tsf_ and 2^nd^γ_ut_.

The strain‐hardening during plastic deformation can be attributed to the SFE and twinning nucleation stress of the materials. Hence, the stacking fault energy (γ_sf_), twinning stacking fault energy (γ_tsf_), unstable stacking fault energy (γ_us_), and second unstable twinning energy (2^nd^ γ_ut_), which represents the energy barriers required to form a stacking fault and nucleate a twin, were estimated by DFT. A two‐layer stacking fault and a three‐layer twin structure were used as shown in **Figure** **8**a. Note that the detailed structure construction process follows the work of Ojha et al.^[^ ^70^ ^]^ The GPFE represents the energy penalty induced by the rigid shift of two parts of the crystal within the BCC (112) plane along the [111] direction.^[^ ^79^ ^]^ To obtain the GPFE curve, γ_us_, γ_sf_, γ_ut_, γ_tsf_, and 2^nd^ γ_ut_, are calculated by:

(1) $$\gamma =\frac{E_{f}\;-\;E_{\mathrm{perf}}}{A_{\left\{112\right\}}}\;$$

where *E*_f_ is the energy of the system with a stacking/twinning fault, *E*_perf_ is the energy of the corresponding bulk system, and A_{112_ is the area of a {112} plane in the simulation cell. During the energy minimization, the atoms in unstable stacking/twinning fault structures are fixed, while the atoms in the stable stacking/twinning fault are free to move. The analytical expression for the twinning stress, developed by Ojha et al.,^[^ ^70^ ^]^ was applied here. The $\gamma_{\mathrm{twin}}^{\prime }$, as defined by Ojha et al.,^[^ ^70^ ^]^ represents the differentiation of *E*_twin_ with respect to position *r*_A_, where *E*_twin_ denotes the energy required to nucleate a twin. Using the fault energies obtained above, the critical twin‐nucleation stress can be estimated as follows:

(2) $$\tau_{\mathrm{critical}}=\frac{1}{b}\;\left\{\gamma_{\mathrm{twin}}^{\prime }-\frac{Gb^{2}}{2\pi }\left(\frac{3-2\sqrt{3}}{\left(\sqrt{3}-1\right)d}\right)\right\}$$

(3) $$\begin{array}{ccc} \gamma_{\mathrm{twin}}^{\prime } & = & \left(\gamma_{\mathrm{ut}}-\frac{\gamma_{\mathrm{ut}}+\gamma_{\mathrm{sf}}}{2}\right)\;\sin\left(2\pi \left[2.5-1.21\right]\right)\\ & & +\frac{1}{4}\left(2^{\mathrm{nd}}\gamma_{\mathrm{ut}}-\gamma_{\mathrm{tsf}}\right)\sin\left(2\pi \left[N-1.22\right]\right) \end{array}$$

(4) $$d=r_{B}\;-r_{A},r_{B}=\frac{3Gb^{2}}{2\pi \gamma_{\mathrm{twin}}^{\prime }},r_{A}=\left(\sqrt{3}-1\right)\;*r_{B}$$

The γ_ut_, γ_sf_, γ_tsf_, and 2^nd^ γ_ut_ are obtained from the GPFE calculation above, *G* is the shear modulus, *b* is the Burgers vector of the twinning dislocation, *r_A_* and *r_B_* are the equilibrium positions of the dislocations on the verge of twinning, and *N* corresponds to the number of layers of the twin nucleus (*N* = 3 in this study).

The mean values of  γ_sf_, γ_tsf_,γ_us_, γ_ut_ and 2^nd^ γ_ut_ for the NbTaTiV RMPEA are γ_sf _= 409.3 ± 49.8 mJ m^−2^, γ_tsf_ = 397.1 ± 58.2 mJ m^−2^, γ_us_ = 584.8 ± 41.4mJ m^−2^, γ_ut _= 578.9 ± 37.7 mJ m^−2^, and 2^nd^ γ_ut _= 528.4 ± 57.4 mJ m^−2^, respectively, as illustrated in Figure 8b. Interestingly, the stacking fault energy and twinning stacking fault energy of the NbTaTiV are smaller than that of its BCC‐phase constituent elements. For example, the γ_sf_ and γ_tsf_ of Nb are 564 and 578 mJ m^−2^, the γ_sf_ and γ_tsf_ of Ta are 592 and 594 mJ m^−2^, while the γ_sf_ and γ_tsf_ of V are 553 and 598 mJ m^−2^.^[^ ^80^ ^]^ The predicted mean critical twin nucleation stress (τ_critical_) for the NbTaTiV is 955.0 ± 121.3 MPa, which is higher than that of composed elements (Nb ≈ 232 MPa, Ta ≈ 231 MPa, and V ≈ 220 MPa).^[^ ^81^, ^82^ ^]^ In contrast to conventional BCC‐phase alloys, in which a lower stacking fault energy generally corresponds to a lower twinning stress, the NbTaTiV exhibits relatively low stacking fault energies with a high twinning stress. This unusual correlation between the SFE and twinning stress is considered to be caused by the large energy differences between the stable and unstable stacking faults. For example, the (γ_ut_ − γ_sf_) of NbTaTiV, Nb, and Ta are 169.6, 61, and 53 mJ m^−2^, respectively, which could result in a higher τ_critical_, according to Equations (2) and (3).^[^ ^83^ ^]^ This trend indicates that one key factor influencing the twinning stress is the energy barrier required to overcome during the twinning nucleation process. Thus, an increase in energy difference between stable and unstable stacking faults could give rise to an increase in twinning nucleation stress.

## 4 Conclusions

A systematic study was performed to understand the deformation behavior of the NbTaTiV as a function of strain‐rate and temperature. Compared to other conventional alloys, the present RMPEA maintains yield strength at high temperatures and shows less strain‐rate sensitivity at both quasi static and high strain rates. Specifically, the strain‐rate sensitivity was measured to be 0.007 along with a thermal softening value of − 0.50. The TEM images from the deformed sample indicated the formation of {112} type‐I thin twins during the high strain‐rate deformation at RT, and it seems that the twins are nucleated within the grain rather than at grain boundaries. Although the presence of twins can lead to an increase in strain‐hardening rate, this feature was not observed in our tests at all temperatures. However, in the current case, the twins were thinner and were all aligned in one direction without any interaction sites. As a result, they could act as barriers to reduce the overall dislocation mobility but also could not be enough for strain hardening to overcome the softening behaviors from adiabatic heating effects.

## 5 Experimental Section

### Material Preparation and Characterization

The NbTaTiV rod samples were manufactured using a vacuum arc‐melting technique with high‐purity elements (99.99 weight % elemental purity), see the detailed experimental procedure listed by Lee et al.^[^ ^3^ ^]^ The fabricated rods were sealed in quartz tube under vacuum and subjected to heat treatment at 1200 °C for 3 d. The samples were water quenched after the heat treatment. ND measurements were performed at RT and 900 °C to identify the crystal structure of NbTaTiV from the VULCAN Engineering Diffractometer at the Spallation Neutron Source (SNS), Oak Ridge National Laboratory (ORNL).^[^ ^84^ ^]^ The microstructure was further characterized, using Inspect –SEM equipped with EBSD. Additionally, planar‐view TEM specimens were extracted from a selected area in the EBSD‐IPF map using a FEI Dual Beam Helios 600 Nanolab. To prepare samples for TEM, rough milling was conducted with an ion current of 65 nA at 30 kV. The ion current was decreased to 80 pA during the polishing process. The surface of the sample was further cleaned using a 5 kV ion beam. The specimens were characterized by employing a Cs‐corrected TEM FEI Titan 80–300 operated at 300 kV.

### Mechanical Tests

Quasi‐static mechanical tests (strain‐rates of 10^−3^, 10^−1^, and 5 s^−1^) under uniaxial compression were performed at varying temperatures, ‐ 187 °C, RT, 300 °C, 500 °C, 700 °C, 800 °C, and 900 °C, using a servo‐hydraulic machine. The samples of 4 mm in diameter and 8 mm in length were used for these tests. Dynamic‐compression experiments were also performed at varying temperatures (RT, 300 °C, 600 °C, and 850 °C), using a Split Hopkinson Pressure Bar (SHPB). Right cylinder samples, 4 mm in diameter and length, were placed in a sealed chamber for high‐temperature mechanical tests. The high‐strain tests were interrupted at varying strains using steel rings of different thicknesses. Microstructure of these samples was then analyzed to understand the deformation behavior in RMPEAs. During the high‐temperature experiments, the test specimens were heated to the target temperature using a furnace integrated with the MTS and Hopkinson Bar test systems. The temperature of the specimens was monitored by attaching a thermocouple directly to the sample surface. The heating rate was maintained at 20 °C per minute but was reduced to 5 °C per minute during the final 50 °C approach to the target temperature to avoid overheating. The mechanical test results are obtained from five and three repeated trials under quasi‐static and dynamic loading, respectively. All values of yield strengths and stress–strain (SS) curves are chosen as the representative data, which indicates the mean values of yield strengths. The standard deviation of repeated measurements was less than 6% (under quasi‐static loading) and 9% (under dynamic loading) and the trends of yield strength and strain hardening were consistent with each set of tests.

### In Situ Neutron Scattering Experiments

The compressive in‐situ neutron diffraction measurements of NbTaTiV under quasi‐static loading at RT were carried out to identify the elastic and plastic deformation behaviors at the VULCAN, Engineering Diffractometer of the SNS, ORNL. The sample size of homogenized NbTaTiV was 4 mm in diameter and 8 mm in length. The neutron diffraction instrument employs the time‐of‐flight (TOF) arrangement, which enables it to cover a wide range of d‐spacings without additional adjustment of samples or detectors. The in‐situ neutron facility is also equipped with two detectors, collecting the diffraction patterns corresponding to the lattice planes that are parallel to the loading and transverse directions, respectively. A 3.5 × 3.5 mm^2^ slit and 2 mm collimators were used to cover a large volume of sample. A constant load‐control mode with a stepwise‐loading sequence was utilized during the measurement of the diffraction patterns. When the stress level reached the yield strength, the control mode was changed from load to displacement‐control modes with an incremental step of 0.2 mm. After that, single‐peak fitting by a VULCAN Data Reduction and Interactive Visualization software (VDRIVE) program was used to analyze the collected data.

### Density Functional Theory calculation

DFT with the projector‐augmented‐wave (PAW) method was used implemented in the Vienna ab initio simulations package (VASP) to do the generalized planar fault energy (GPFE) calculation of the NbTaTiV RMPEA.^[^ ^85^, ^86^ ^]^ The exchange‐correlation is accounted for within the generalized gradient approximation (GGA), using the Perdew‐Burke‐Ernzerhof (PBE) functional.^[^ ^87^ ^]^ The simulation‐cell size is approximately 8Å  ×  9Å  ×  24Å, oriented along $\left[111\right],\left[1\bar{1}0\right],\mathrm{and}\;\left[11\bar{2}\right]$, respectively. A periodic boundary condition (PBC) was applied in all directions. The Special Quasi‐random Structures (SQS) method was applied to create 15 different configurations of the equiatomic NbTaTiV RMPEA.^[^ ^88^ ^]^ The valence electron configurations of each species are described as follows, V: 3p4s3d, Ta: 6s5d, Nb: 4p5s4d, and Ti: 4s3d. The K‐points grid is 4 × 4 × 2, and the cutoff energy for the plane‐wave‐basis set is 450 eV. The convergence criterion was set so that the change in energy between minimization iterations was 10^−6^ eV, and the force on each atom was below 0.01 eV Å^−1^.

## Conflict of Interest

The authors declare no conflict of interest.

## Supporting information



Supporting Information

## Data Availability

The electron microscopy and mechanical test data are available from the corresponding author (Saryu Fensin, saryuj@lanl.gov and Chanho Lee, czl0176@auburn.edu) upon request. The neutron diffraction data are available from SNS, ORNL, (Ke An, kean@ornl.gov).
