# Supplementary material for: Deformation Behaviors in Single BCC‐Phase Refractory Multi‐Principal Element Alloys under Dynamic Conditions
Source: Adv Sci (Weinh). 2025 Aug 4;12(36):e08180. doi: 10.1002/advs.202508180 (PMC12463061; doi:10.1002/advs.202508180)
Supplement: Supplementary file 1 — Supporting Information [file ADVS-12-e08180-s001.pdf]

ADVANCED SCIENCE
Open Access

Supporting Information

for *Adv. Sci.*, DOI 10.1002/advs.202508180

Deformation Behaviors in Single BCC-Phase Refractory Multi-Principal Element Alloys under Dynamic Conditions

Chanho Lee, Deva Prasaad Neelakandan, Dongyue Xie, Juntan Li, Chia-Yi Wu, Aomin Huang, Leeseung Kang, Shuozhi Xu, Barton C. Prorok, Dong-Joo Kim, Marc A. Meyers, Haixuan Xu, Peter K. Liaw, Yi-Chia Chou, Ke An, George T. Gray III, Nan Li, Gian Song* and Saryu J. Fensin**

Supporting information for the manuscript

Deformation behaviors in Single BCC-Phase Refractory Multi-Principal Element Alloys under Dynamic Conditions

Chanho Lee*, Deva Prasaad Neelakandan, Dongyue Xie, Juntan Li, Chia-Yi Wu, Aomin Huang, Leeseung Kang, Shuozhi Xu, Barton C. Prorok, Dong-Joo Kim, Marc A. Meyers, Haixuan Xu, Peter K. Liaw, Yi-Chia Chou, Ke An, George T. Gray III, Nan Li, Gian Song*, and Saryu J. Fensin*

C. Lee, D.P. Neelakandan, B.C. Prorok, D.J. Kim
Materials Engineering, Auburn University, Auburn, AL 36849 USA
Email: czl0176@auburn.edu

D. Xie, G.T. Gray III, N. Li, S. J. Fensin
Center for Integrated Nanotechnologies, MPA Division, Los Alamos National Laboratory, Los Alamos, NM 87545, USA
Email: saryuj@lanl.gov

J. Li, H. Xu, P. K. Liaw
Department of Materials Science and Engineering, The University of Tennessee, Knoxville, TN 37996-2100, USA

C. Wu, Y. Chou
Department of Electrophysics, National Yang Ming Chiao Tung University, Hsinchu 30010, Taiwan

A. Huang, M.A. Meyers
Department of Mechanical and Aerospace Engineering, University of California, San Diego, CA 92093, USA

L. Kang
Korea Institute for Rare Metals, Korea Institute of Industrial Technology (KITECH), Incheon, 21655, Republic of Korea

S. Xu
School of Aerospace and Mechanical Engineering, University of Oklahoma, Norman, OK 73019-1052, USA

K. An
Neutron Scattering Division, Oak Ridge National Laboratory, Oak Ridge, TN 37831, USA

G. Song
Division of Advanced Materials Engineering, Kongju National University, Cheonan, Chungnam, 330-717, Republic of Korea
Email: gasong@kongju.ac.kr

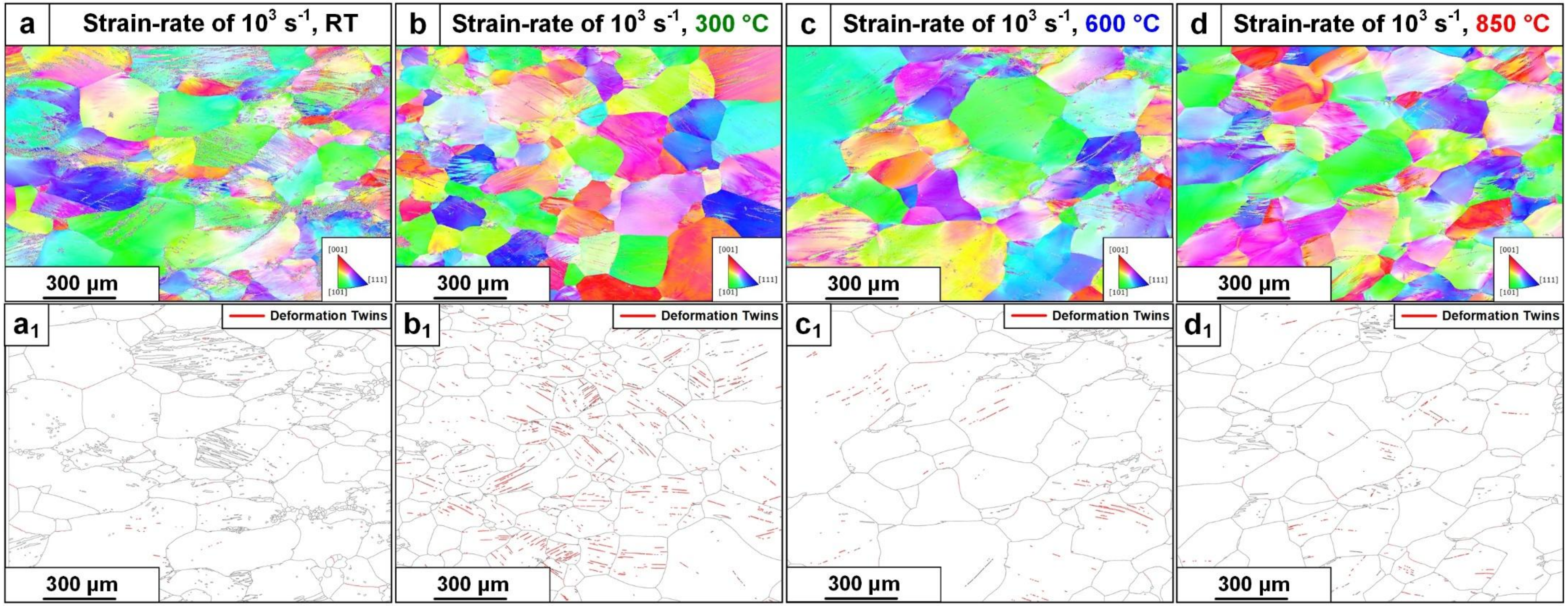

Figure S1. The EBSD-IPF and deformation twin images obtained from a 15 %-deformed NbTaTiV at elevated temperature. (a, a_1) RT, (a, b_1) 300 °C, (c, c_1) 600 °C, and (d, d_1) 850 °C after deformation at a strain-rate of 10^3 s^{-1}.

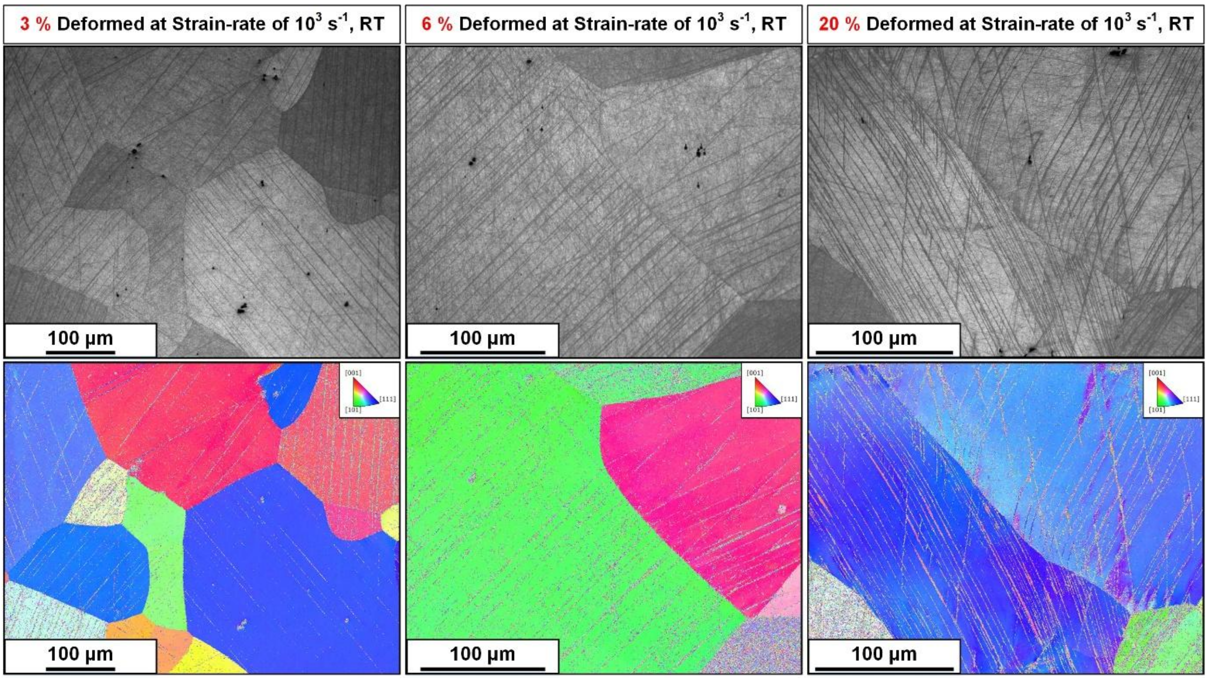

Figure S2. Observation of twin thickness evolution as a function of plastic strain. Microstructural evolutions of NbTaTiV as a function of plastic strains (3, 6, and 20 %) at a strain-rate of 10^3 s^{-1} and RT.

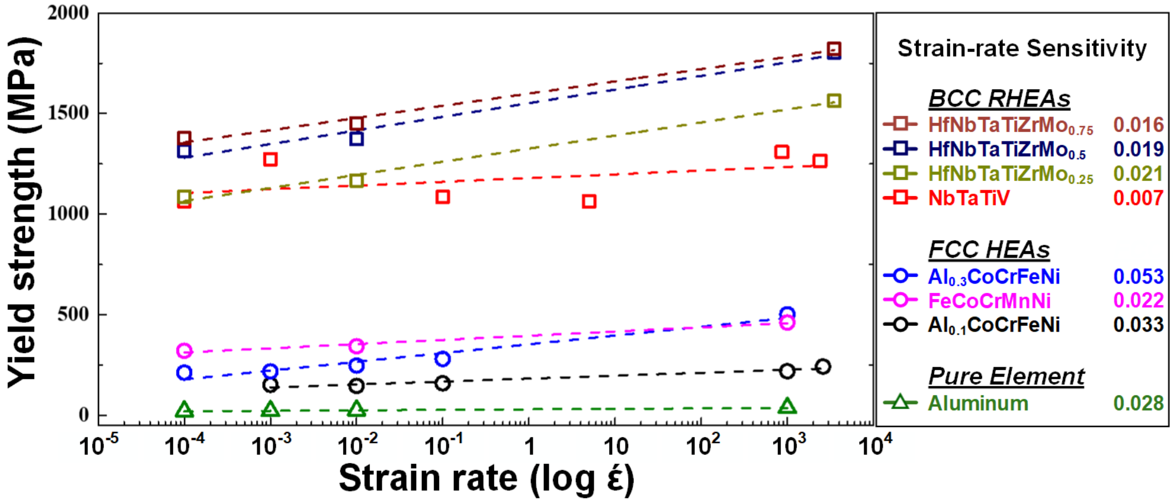

Figure S3. Comparison of strain-rate sensitivity of different alloys. Evolution of yield stress as a function of logarithmic strain-rate for BCC-phase RMPEAs (HfNbTaTiZrMo and NbTaTiV), FCC-phase MPEAs (AlCoCrFeNi and FeCoCrMnNi), and pure aluminum metal.
